# Electrical interface design for additively manufactured polymer anodes for biophotovoltaic systems

**DOI:** 10.3389/fmicb.2026.1858659

**Published:** 2026-05-21

**Authors:** Maira Anam, Geoffrey Rivers, Rachel Louise Gomes, Ricky Wildman, Helena I. Gomes

**Affiliations:** 1 Food Water Waste Research Group, Faculty of Engineering, University of Nottingham, University Park, Nottingham, United Kingdom; 2 Centre for Additive Manufacturing, Faculty of Engineering, University of Nottingham, Jubilee Campus, Nottingham, United Kingdom

**Keywords:** 3D printing, bioelectrochemical systems, PEDOT:PSS, cyanobacteria, electrode

## Abstract

**Introduction:**

Reliable electrical interfacing between additively manufactured conductive polymer anodes and external circuits remains a key challenge in biophotovoltaic (BPV) systems, particularly due to debonding failures during operation. This study aims to identify interface configurations that enhance mechanical stability and electrical performance while maintaining biocompatibility, thereby improving device reliability.

**Methods:**

A comprehensive evaluation was conducted on multiple electrical connection strategies for inkjet-printed poly(3,4-ethylenedioxythiophene) polystyrene sulfonate (PEDOT:PSS) anodes. Mechanical connection approaches tested included tantalum wire clips, terminal block connectors, crocodile clips, and carbon tapes. In parallel, conductive adhesive materials—silver conductive paint, electric paint, and carbon cement adhesive—were assessed. Each interface was systematically evaluated for mechanical durability, electrical resistance, and biocompatibility in a water-based growth medium. Protective coatings were also applied to selected adhesive interfaces to improve performance.

**Results:**

The findings show that mechanical connection methods are unsuitable for inkjet-printed PEDOT:PSS anodes, as they can damage the polymer film and are prone to corrosion. In contrast, adhesive-based interfaces demonstrated improved compatibility but required protective coatings to enhance durability. Among the tested materials, silver conductive paint coated with epoxy resin showed the best performance, achieving 100% stability in aqueous medium over 7 days. Transparent silicone resin coatings provided moderate improvement, achieving 33.4% stability. Overall, the optimized configurations reduced in-operation debonding failure from 66% to less than 1%.

**Discussion:**

These results highlight the critical role of interface selection and protection strategies in improving the longevity and performance of BPV systems. Adhesive-based electrical interfaces, when combined with appropriate protective coatings, offer a viable solution for maintaining stable connections with delicate conductive polymer anodes. The framework established in this study provides a systematic basis for assessing electrical interface materials, contributing to the optimization of electrode connectivity and overall device efficiency in BPV applications.

## Highlights

- First report of electrical interface materials for inkjet-printed PEDOT:PSS anodes
- Mechanical connectors physically damaged inkjet-printed PEDOT:PSS film
- Adhesives bonding interfaces required protective overcoats to achieve long-term stability
- Systematic framework to select robust electrical interface for future BPV studies

## 1 Introduction

Biophotovoltaic (BPV) use photosynthetic microorganisms (algae and cyanobacteria) or their sub-cellular components to generate electricity directly from sunlight (Zhu et al., 2023). The fundamental architecture includes an anode, cathode, membrane, and electrical circuit components (de Fouchécour et al., 2022). Despite the promising potential of BPVs as a sustainable power generator, the reported energy conversion efficiency is lower (< 1%) than the calculated theoretical maximum efficiency value (2.9% of initial solar input) (McCormick et al., 2015). Not only are BPV prone to inherent intrinsic energy losses of bioelectrochemical systems that could potentially occur during operation (Logan et al., 2006), but the electron transfer networks in photosynthetic microbes are, in general, not adapted for exoelectrogenic activity (McCormick et al., 2011). The current BPV system's failure to achieve the theoretically estimated power outputs indicates a need for improvement. The ohmic and activation losses in the anodic and cathodic compartments are sensitive to system operating parameters and structural components such as the electrical interface. In BPV, the role of the electrical interface component is to connect the electrode with the wires of the external circuit and facilitate the flow of electrical current (Paitier et al., 2022; Jung et al., 2018). Detailed reviews of biophotovoltaic system design, performance metrics and anode materials can be found in the literature (Bombelli et al., 2012; Tschörtner et al., 2019; Anam et al., 2021).

The rational selection and design of the electrical interface between the anode and the external circuit is crucial for maximizing the performance efficiency of a BPV. An ideal electrical interface for BPV requires electrical conductivity, mechanical robustness in a liquid medium immersion, electrochemical stability in various environmental conditions, corrosion resistance, low electrical resistance with electrode material, and either biocompatibility or material containment (Anam et al., 2021). In most BPV systems, electrical connections are typically established via conductive substrates or leads positioned outside the electrolyte, as implied by the reported device architectures and measurement configurations, thereby avoiding direct aqueous exposure of the electrical interface (Chen et al., 2025; Wey et al., 2024). In general, issues related to aqueous instability and corrosion can be mitigated by positioning the electrical interface outside the electrolyte, as electrical connections may be established either within or external to the electrolyte, depending on reactor design and operational considerations. However, this requires appropriate adaptation of the reactor's architecture. The present study focuses explicitly on the development of electrical contacts formed within the main body of BPV system. Despite the role of the interface material in electron transport at the electrode interface being well acknowledged, insufficient attention has been given to the targeted design strategies for electrical interface material, and the problem remains largely unexplored in the literature (He et al., 2021; Cornejo et al., 2018). The choice of material and design of the electrical interface can greatly influence the performance of the BPV system. A systematic approach to designing and testing electrical interface material would help identify promising candidates that decrease interface resistance, improve electrical conductivity, and offer reasonable mechanical support and biocompatibility (Anam et al., 2021).

Poly (3,4-ethylene dioxythiophene) polystyrene sulfonate (PEDOT:PSS) is an electrically and ionically conductive polymer, commonly used in optoelectronic devices due to its optical transparency and electrical conductivity (Mengistie et al., 2014). Despite its widespread adoption in the optoelectronics (Fan et al., 2023), the application of PEDOT:PSS as an anode material in BPV has not been extensively explored in the existing literature, with few exceptions (Chen et al., 2025; Liu and Choi, 2017; Anam et al., 2026). Consequently, the carbon-based and thin film (indium tin oxide and fluorine-doped tin oxide deposited on plastic substrate) anode remains the most extensively used in BPV research (Bombelli et al., 2012; Thong et al., 2022; Ng et al., 2014). The successful integration of PEDOT:PSS to BPV technology requires the understanding and selection of feasible fabrication technology. Inkjet printing is an additive manufacturing technique used to deposit liquid phase material onto a substrate digitally controlled by computer-aided design (Mesforush et al., 2025). Compared with conventional thin-film fabrication methods such as spray-coating, spin-coating, vapor-liquid phase deposition and electrodeposition, inkjet printing offers high accuracy and resolution for printing microstructures (Zhang et al., 2024). The other advantages of inkjet printing include broad substrate compatibility, patterning flexibility, rapid fabrication, digital reproduction of print, printing feasibility across a relatively large working area, pinpoint accuracy, and ink conservation in a tightly controlled fashion (Yang and Yu, 2025). This makes inkjet printing ideal for printing electronic devices (Magdassi, 2009). In our previous study, novel insight was put toward the adoption of inkjet-printed PEDOT:PSS anode in BPV system. It was noted that inkjet-printed PEDOT:PSS film on UV-ozone treated PET substrate outperformed indium tin oxide coated PET as an anode, although trade-offs exist among the desired optical transparency and electrical resistance, number of printed layers and microbial occupancy (Anam et al., 2026).

In this study, different interface materials and configurations were systematically evaluated for integrating inkjet-printed poly(3,4-ethylenedioxythiophene) polystyrene sulfonate (PEDOT:PSS) as an anode in a biophotovoltaic system. The objective was to identify an electrical conductivity and mechanically durable interface capable of withstanding prolonged immersion in aqueous growth medium without causing adversity to microbial biofilm. Accordingly, the tested interfaces were subjected to a sequential screening process involving mechanical robustness, aqueous aging stability, electrical conductivity, and preliminary microbial growth compatibility. This study offers a practical materials-selection framework for electrical contacts in submerged BPV architectures. The details related to additive manufacturing of inkjet-printed PEDOT:PSS and photocurrent generated using the respective anode can be found here (Anam et al., 2026).

## 2 Materials and methods

### 2.1 Inkjet-printing PEDOT:PSS anode

Poly(3,4-ethylenedioxythiophene):polystyrene sulfonate (PEDOT:PSS) based polymer anodes were fabricated via inkjet printing using an ink formulation and printing parameters adapted from previous work (Rivers et al., 2023). Briefly, PEDOT:PSS ink was formulated by mixing 32 wt.% of PEDOT:PSS aqueous solution (Clevois PH1000 1.1 wt.%) with 4 wt.% Cyrene, 0.5 wt.% Glycerol carbonate, 0.2 wt.% Polysorbate 80, 3.7 wt.% Butanol, 0.3 wt.% (3-glycidyloxypropyl) trimethoxysilane, and 59.3 wt.% deionised water. The mixture was magnetically stirred at 1200 rpm overnight, subsequently printed on UV-ozone treated glass and plastic film (PET) using a piezoelectric inkjet printer, Fujifilm Dimatix (DMP 2831) (Anam et al., 2026). The quality of the printed sample was observed with a light microscope (Nikon Eclipse LV100ND, reflection mode, 5x and 10x).

### 2.2 Culturing of *Synechocystis* sp. PCC 6803 cyanobacteria

*Synechocystis* sp. PCC 6803 glucose sensitive strain was cultured autotrophically in standard blue-green 11 (BG11, pH 8.0) medium by adding stock solutions and trace element solution (Supplementary Tables S1, S2) to deionised water (Milli-Q^®^) following guidelines previously established (Moghazy, 2019). The media was supplemented with 10 mM sodium bicarbonate. The cultures were grown in aerated Nunc™ EasYFlask™ at 30 °C under constant irradiance of 60 μEm^−2^s^−1^ and shaking (150 rpm, Infors shaking incubator). The growth was monitored by measuring optical density at 750 nm (OD_750_) after every 24 hours using Agilent 8453 UV-vis spectrometer.

### 2.3 Electrical interface testing

**Figure 1:**
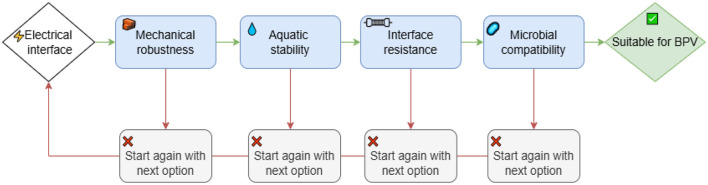
Sequential assessment process for selecting suitable electrical interface materials for BPV systems, including mechanical, chemical, electrical, and biological screening steps.

Eight electrical junction configurations: five mechanical and three adhesive interface methods were evaluated for their mechanical stability, electrical resistance, and biocompatibility under both dry and aqueous conditions. The mechanical interface options included tantalum wire clips (Redox.me), terminal block connectors (RS PRO), copper foil tape (RS PRO), carbon tape (RS PRO), and miniature brass crocodile clips (Hirschmann AGF 1, 1 mm clamping range). The adhesive interface materials tested were electric paint (Bare Conductive^®^, Supplementary Table S3), carbon cement adhesive (Thermo Scientific™, Supplementary Table S4), and silver conductive paint (RS PRO, Supplementary Table S5). Each adhesive interface was further reinforced with a non-conductive protective overcoat surface mount adhesive epoxy resin (RS PRO, Supplementary Table S6), silicone conformal coating (Electrolube^®^, Supplementary Table S6), or two-part rapid epoxy (Araldite^®^, Supplementary Table S6) to enhance mechanical durability. Electrical junctions were established between titanium wires (Diameter: 0.25 mm, 99.6% purity, Goodfellow Cambridge Ltd.) and the printed electrode material using each of these specified interface methods. All experiments were conducted in triplicate (number of samples = 3). The following test sequence was used for the electrical interface under laboratory conditions (Figure 1).

i. ***Mechanical stability:*** The mechanical stability of the electrical interface between the inkjet-printed PEDOT:PSS anode and the external circuit was visually assessed by manually oscillating (waving) it in the air along the x- and y-axes for 15 seconds under dry conditions. For consistency, mechanical failure under dry handling was defined as any visible crack formation in the conductive joint, loosening of the titanium wire from the substrate, complete or partial detachment of the conductive adhesive from the printed electrode region after manual oscillation. While this preliminary assessment provides an initial indication of connection robustness, detailed mechanical stability was further evaluated through delamination analysis to obtain more quantitative insights.
ii. ***Aqueous stability:*** The mechanical stability under aqueous conditions was assessed by immersing the interface assembly (inkjet-printed PEDOT:PSS anode + selected interface option + titanium wire) in BG11 growth medium for a minimum of 24 h at 30 °C under continuous illumination (60 μE m^−2^ s^−1^). For the optimized adhesive interfaces, prolonged immersion was extended to 7 days under the same operational conditions. Samples were inspected periodically (24 h, 72 h, 120 h, and 168 h) and failure was defined as the occurrence of one or more of the following: (i) visible delamination of the conductive joint from the substrate, (ii) crack propagation within the adhesive region, (iii) swelling/softening of the protective overcoat, (iv) dissolution/discolouration of conductive adhesive into the medium, or (v) loss of electrical continuity. Representative photographic images were recorded to document physical changes. For interfaces involving copper tape, a copper cuvette test kit (HACH^®^, LCK 329, 0.1–8.0 mg/L) was employed to quantify copper ion release into the BG11 medium.
iii. ***Interface stability:*** For selected stable interface configuration, the two-probe resistance was measured with a digital Multimeter (RS PRO). Measurements were performed in triplicate at room temperature.
iv. ***Biocompatibility***: The biocompatibility was evaluated by growing *Synechocystis* sp. PCC 6803 in the presence of the selected electrical interfaces. The sample (inkjet-printed PEDOT:PSS anode + selected interface option + titanium wire) was suspended inside the 50 mL Nunc™ EasYFlask™ containing *Synechocystis* sp. PCC 6803 glucose sensitive (Rippka et al., 1979) strain culture inoculated at an OD_750_ of 0.1 in 20 mL of BG11 medium. The culture was grown at 30 °C under the illumination of 60 μEm^−2^s^−1^ and 150 rpm agitation. Microbial growth was monitored spectrophotometrically by measuring optical density at 750 nm (OD_750_) using a Shimadzu UV2600 spectrometer. In the present study, OD_750_ was employed as an initial screening indicator to identify gross growth inhibition or acute cytotoxicity caused by the selected interface materials. More comprehensive physiological indicators such as chlorophyll fluorescence, oxygen evolution, cell viability staining, and extracellular electron transfer analysis were not included at this stage and should be considered in future studies.

To verify the practical applicability of the selected electrical interface, the silver paint/surface-mount epoxy/titanium wire assembly was incorporated into a laboratory-scale biological photovoltaic (BPV) system following the configuration previously reported (Anam et al., 2026). Briefly, a customized single-chamber cubic BPV reactor was used integrating a platinum-coated gas diffusion electrode (0.5 mg cm^−2^ Pt on Vulcan carbon cloth, Fuel Cell Store, USA) cathode and inkjet-printed PEDOT:PSS anode connected via the selected electrical interface serving as the anode. *Synechocystis* sp. PCC 6803 cells were cultured in BG11 medium and immobilized onto the anode prior to assembly. BG11 growth medium was used as the electrolyte, and BPVs were operated at 30 °C under continuous white light of intensity 60 μEm^−2^s^−1^. Electrochemical characterization of BPVs was carried out using a Metrohm Autolab PGSTAT204 potentiostat (with NOVA software version 2.1). Data processing and analysis were performed using Metrohm NOVA software. Ag/AgCl electrode (BASi MW-2030) was used as reference. Cyclic voltammetry was conducted for interface made with inkjet-printed PEDOT:PSS anode within the potential window of 1 to −1 V at a scan rate of 100 mVs^−1^. The electrochemical impedance spectroscopy was performed to measure the internal resistance of BPV, and the frequency of the alternating signal was varied from 100 Hz to 100 KHz with an amplitude of 10 mV.

## 3 Results and discussion

**Figure 2:**
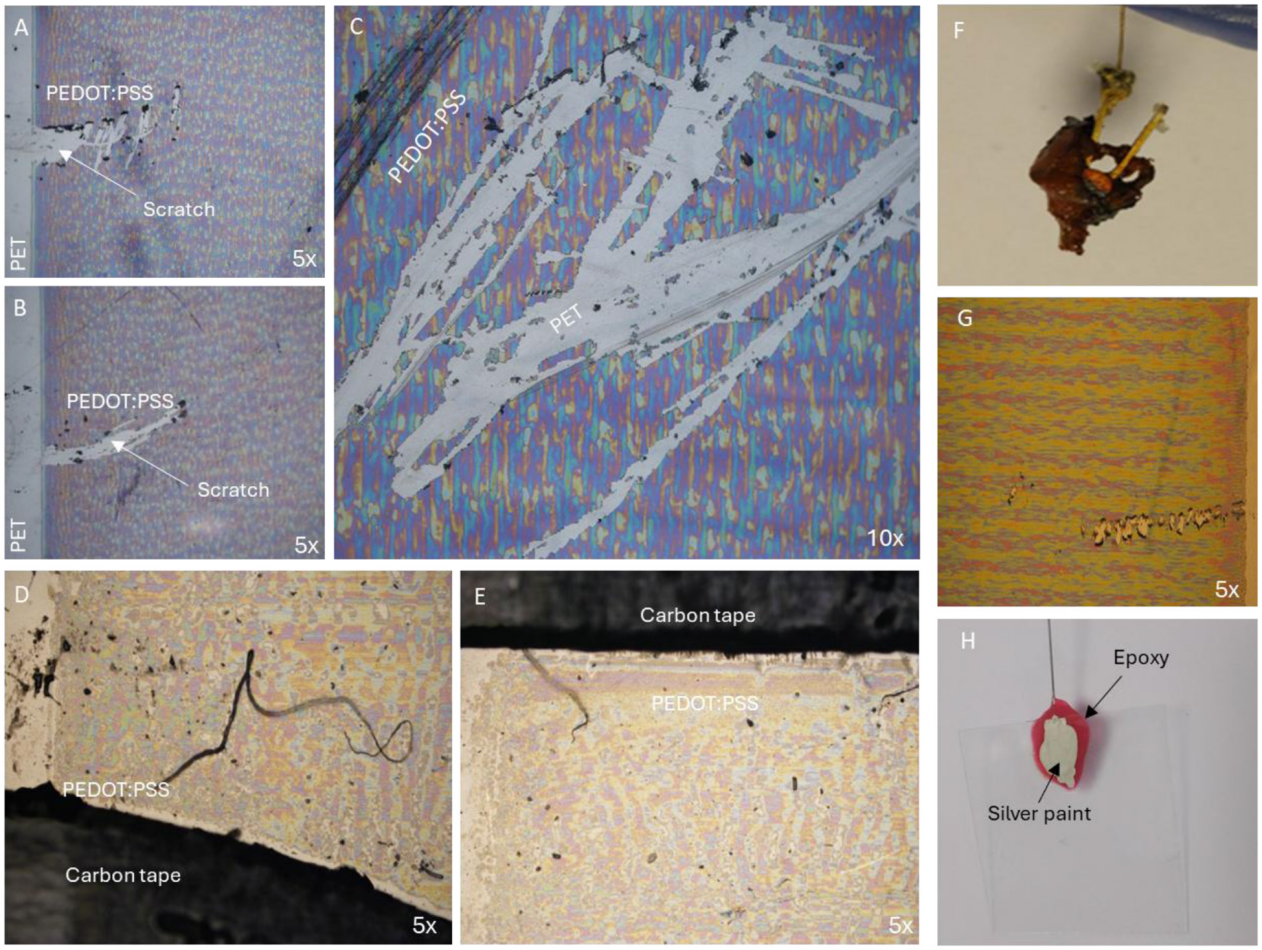
Electrical interface configurations evaluated in this study. **(A–C)** Optical microscopy images showing mechanical damage to inkjet-printed PEDOT:PSS (6 layers) induced by a tantalum wire clip. **(D, E)** Optical microscopy images of PEDOT:PSS inkjet-printed around carbon tape, revealing a visible interfacial gap (black region) and non-uniform film formation due to poor wetting on the substrate. **(F)** Corrosion observed on a crocodile clip–titanium wire assembly after 24 h incubation in BG11 medium. **(G)** Optical microscopy image showing mechanical damage to PEDOT:PSS film caused by the crocodile clip. **(H)** Silver conductive paint contact protected with an epoxy overcoat (surface-mount adhesive epoxy resin) connected to a titanium wire.

The tantalum wire clip evaluated as an electrical interface for inkjet-printed PEDOT:PSS anode was unable to securely hold the glass or plastic substrates used. While these clips might be suitable for the anode with fibrous texture, such as carbon paper (Thickness: 0.25 mm), the thickness of glass slide (170 μm) and plastic film (74 μm) made them incompatible with the clip's opening dimensions (Table 1). Additionally, the pressure exerted during the assembly caused mechanical damage to the PEDOT:PSS deposited layer (Figures 2A–C), compromising the anode's structural integrity.

**Table 1:** Summary of electrical materials tested in this study with inkjet-printed PEDOT:PSS anode.

Electrical contact material	Mechanical stability	Aquatic stability	Interface resistance	Biocompatibility
Tantalum wire clip (Redox.me)	x	–	–	–
Terminal block connector (RS PRO)	✓	x	–	–
Copper tape (RS PRO)	✓	x	–	–
SEM carbon tape (RS PRO)	x	–	–	–
Crocodile clip (Hirschmann AGF 1)	✓	x	–	–
Silver paint (RS PRO)	x	–	–	–
Silver paint (RS PRO) + Red Syringe Surface Mount adhesive epoxy resin (RS PRO)	✓	✓	✓	✓
Silver paint (RS PRO) + 2-part rapid epoxy (Araldite^®^)	✓	x	–	–
Silver paint (RS PRO) + Transparent Silicone Resin conformal coating (Electrolube^®^)	✓	x	–	–

Conductive carbon adhesive tape was also examined as an alternative interface. In this configuration, carbon tape was applied to the plastic substrate, followed by the inkjet printing of the PEDOT:PSS film overlapping the tape edge. Poor film uniformity was observed due to adhesive residues that impeded wetting at the tape-film interface. The microscopic examination revealed discontinuities between the PEDOT:PSS layer and the carbon tape (Figures 2D, E), attributed to poor wetting of the ink in regions contaminated with tape adhesive residue. The residues likely inhibited uniform film spreading and adhesion.

Similarly, conductive copper tape was tested as an electrical interface. However, after 24 hours of immersion in BG11 growth medium, copper concentration increased in the BG11 medium (from 0.0084 mg L^−1^ to 0.386 mg L^−1^), indicating galvanic corrosion and potential copper toxicity (Zhen et al., 2021), and were therefore excluded from further analysis. Another attempt employed a miniature brass crocodile clip spot-welded with titanium wire to form an electrical junction. This configuration was vertically submerged inside BG11 medium and incubated at 30 °C for 24 h. Visible corrosion was observed within 4 h of incubation, particularly around the spring region, and progressively intensified over time (Figure 2F). This finding is noteworthy given the frequent use of such clips in bioelectrochemical research (Barrena et al., 2008). When applied to inkjet-printed PEDOT:PSS films, the crocodile clip caused visible damage and disruption of the conductive layer (Figure 2G), rendering it unsuitable for inkjet-printed PEDOT:PSS anode.

For adhesive bonding approaches, titanium wires were connected to the substrate using electric paint by dipping the wire into the paint and positioning it at the substrate edge, similarly to what has been done in literature (Kasim et al., 2020; Tanwilaisiri and Kajondecha, 2021). These trials were performed on UV-ozone treated glass and PET substrates without PEDOT:PSS coatings to evaluate paint-substrate interaction. Despite initial adhesion, the water-susceptible formulation of electric paint (Supplementary Table S3) dispersed in the BG11 medium within 24 h, turning the solution black, indicative of its poor cohesive stability in aqueous conditions. Another attempt to establish an electrical interface was made by using carbon conductive cement adhesive (Supplementary Table S4). A small droplet of the adhesive was placed on both untreated and UV-ozone treated glass and PET substrates, connecting them to a titanium wire. The assemblies were air-dried overnight and subsequently incubated in BG11 medium and deionised water for 24 h at 30 °C. In all cases, the adhesive bond failed, with complete delamination of the junction from the substrate, indicating insufficient adhesion strength for aqueous applications.

Silver conductive paint, a low viscosity colloidal suspension of silver particles (average size: < 10 μm), was also evaluated as an interface material (Supplementary Table S5). This paint is designed to infiltrate narrow interfacial gaps between conductive surfaces to form robust electrical connections (Wang et al., 2007). Titanium wires were attached to the glass and PET substrates by coating with silver paint and allowing the assemblies to air-dried overnight prior to immersion in BG11 medium. The silver paint failed to establish a mechanically durable joint on both PET and UV-ozone treated glass substrate under immersion. To enhance mechanical durability, a non-conductive overcoat was applied on top of the silver paint, extending slightly beyond the interface to anchor the connection to the substrate (Figure 2H), as previously demonstrated (Wang et al., 2007). Three overcoating materials were tested: (i) Surface mount adhesive epoxy resin, (ii) Transparent silicone resin conformal coating, and (iii) 2-part rapid epoxy (Supplementary Table S6). The stability assessment was based on prolonged immersion tests under operational conditions (BG11 medium, 60 μEm^−2^s^−1^, 30 °C, pH: 8 for 7 days), during which no delamination, cracking, swelling, or loss of electrical continuity was observed.

**Figure 3:**
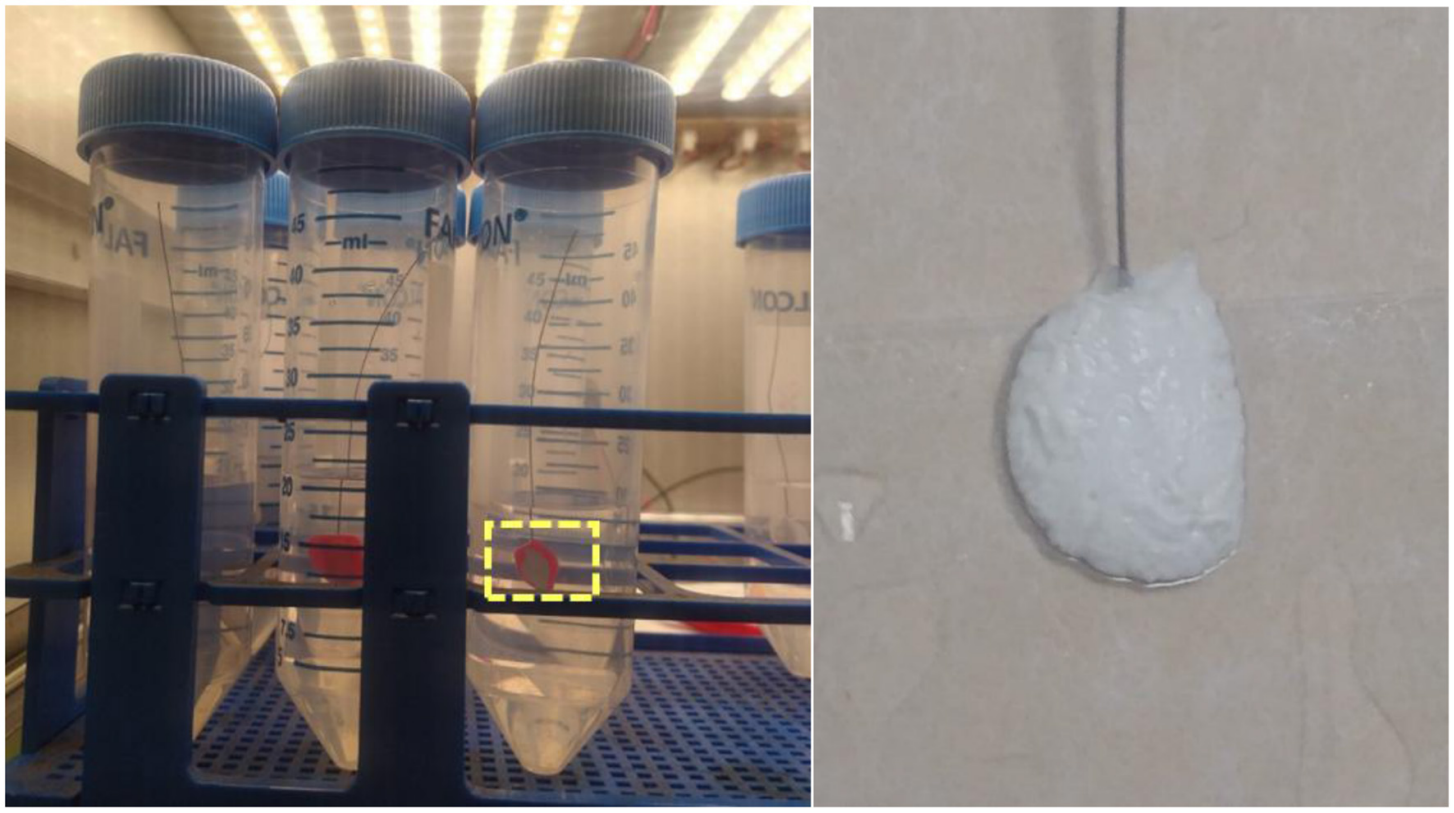
Silver paint with an epoxy overcoat assembly incubated in BG11 medium **(left)** Araldite^®^ epoxy overcoat on silver paint after 24 h of incubation in BG11 medium **(right)**.

The overcoating material improved the mechanical robustness of the silver joints by reinforcing the connection and preventing direct exposure to the medium, the interface joint failed on the PET substrate after aqueous aging in BG11 medium (Failure percentage: 100%, *n*= *3*). In contrast to the PET substrate, some interfaces on UV-ozone treated glass remained intact in BG11 medium for a period exceeding 5 days (Salley et al., 2012). Among the three non-conductive overcoat materials examined (Table 2), the transparent silicone conformal coating exhibited the highest susceptibility to aqueous failure, with two out of three samples showing progressive softening and interfacial detachment during immersion (failure rate: 66.6%). In contrast, both the surface-mount adhesive epoxy resin and the two-part rapid epoxy retained physical attachment throughout the 7-day aging period with no complete delamination observed (0% failure, *n* = 3). However, despite retaining adhesion, the two-part rapid epoxy underwent visible whitening and surface wrinkling after prolonged immersion, suggesting water-induced structural alteration of the polymer matrix (Figure 3). The surface-mount adhesive epoxy resin showed the highest dimensional and visual stability, with no observable swelling, crack formation, surface wrinkling, or loss of wire anchorage over the 7-day test period. For this reason, it was selected as the most suitable protective overcoat for subsequent testing. The distinct behavior of the tested interface materials highlights the importance of simultaneous conductive adhesion and environmental sealing in submerged BPV applications. Silver paint alone provided electrical contact but formed only a thin particulate conductive bridge with limited mechanical anchorage to the substrate, making it vulnerable to adhesive failure upon physical stress and hydration. Application of an epoxy overcoat redistributed local mechanical stress around the titanium wire, increased the effective bonded area, and reduced direct penetration of the aqueous medium into the conductive junction. The inferior performance of the silicone conformal coating may further reflect its higher elasticity and permeability to water ingress, which can progressively weaken the conductive underlayer.

**Table 2:** Aquatic stability of three different non-conductive overcoat materials.

Epoxy overcoat	Failure percentage
Surface mount epoxy resin	0%
Silicone conformal coating	66.6%
2-part rapid epoxy	0%

Failure percentage was calculated using Failure percentage = R/T × 100, where R is the number of failures, and T is the total number (Number of samples tested, *n* = 3).

In addition to retention of the physical joint during aqueous aging, the silver paint/surface-mount epoxy interface preserved measurable electrical continuity following immersion in BG11 medium. Two-probe measurement performed on the intact assemblies after the aqueous stability screening yielded an average interfacial resistance of 15.5 ± 1.0 kΩ (*n* = 3), confirming the persistence of a conductive pathway between the inkjet-printed PEDOT:PSS layer and the titanium wire. It is to be noted that this measurement does not resolve resistance evolution during immersion, it demonstrates that the interface remained electrically connected after prolonged aqueous exposure. Although this interfacial resistance is substantially higher than the intrinsic sheet resistance of the printed PEDOT:PSS film (29.5 Ωsq^−1^) (Anam et al., 2026), it is consistent with the expected behavior of manually assembled particulate conductive adhesive joints. The resistance is governed not by the bulk conductivity of silver itself but by the active contact area, particle percolation pathways, and the presence of microscale voids (air gaps) at the wire–adhesive–polymer interface (Wang et al., 2007). Therefore, the measured kilo-ohm resistance primarily reflects contact limitations of the assembled junction rather than the conductivity of the silver paint and PEDOT:PSS anode material. Further investigation is therefore required to evaluate the interfacial morphology and minimize such defects.

**Figure 4:**
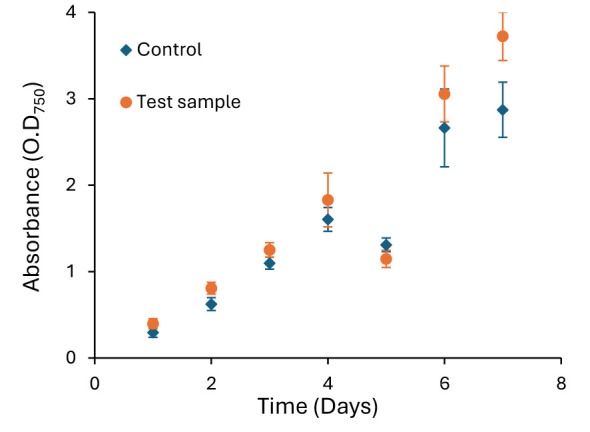
*Synechocystis* sp. PCC 6803 growth curve in the presence of electrical interface material (wire + silver paint + surface mount epoxy) and control (no electrical interface material). Average data is presented here with standard deviation (triplicate experiments).

*Synechocystis* sp. PCC 6803 cultivated in the presence of the assembled interface exhibited OD_750_ values statistically comparable to the control culture without interface materials (*p* > 0.05), indicating the absence of acute growth suppression over the cultivation period (Figure 4). This observation suggests that no immediate leachable toxicity or severe incompatibility was introduced by the encapsulated silver–epoxy interface under the tested conditions. However, it should be noted that OD_750_ provides only a bulk biomass estimate and does not fully resolve cellular physiological stress, photosynthetic efficiency, or extracellular electron transfer behavior. Thus, further in-depth investigation is needed to conclusively confirm biocompatibility of the silver-epoxy interface.

### 3.1 Biological photovoltaic system

**Figure 5:**
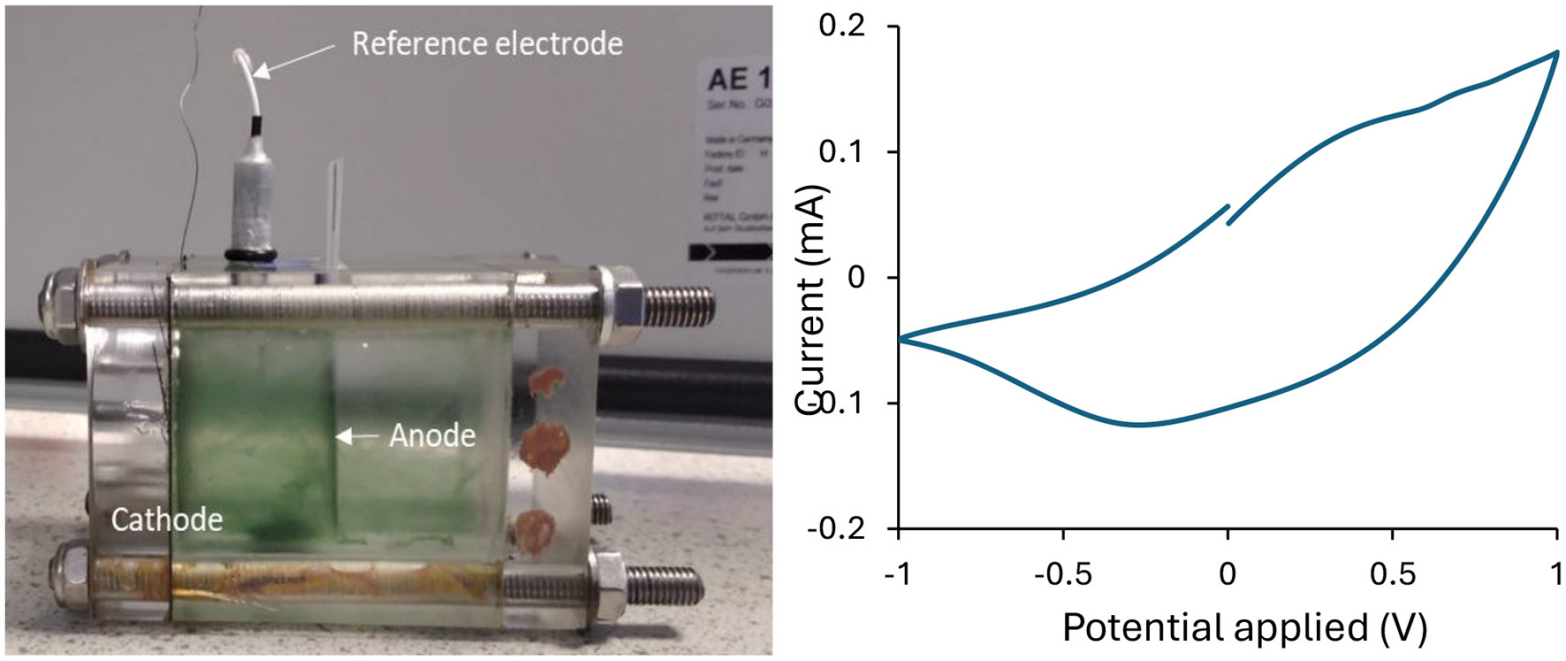
Biological photovoltaic system used in this study **(left)**. Cyclic voltammogram of inkjet-printed PEDOT:PSS anode in BPV system with silver–epoxy interface at the scan rate of 100 mVs^−1^ **(Right)**.

To evaluate that the silver paint/surface-mount epoxy interface remained functional beyond benchtop immersion screening, the same configuration (silver paint/surface-mount epoxy/titanium wire) was integrated into an operating BPV reactor (Figure 5). Under continuous illumination, the assembled PEDOT:PSS-based BPV generated a measurable maximum power density of 0.013 mW cm^−2^, thereby validating the practical suitability of the electrical interface for submerged BPV application. Detailed reactor configuration, long-term photocurrent behavior, and comparative BPV performance metrics are provided in the previous study (Anam et al., 2026).

Cyclic voltammetry exhibited a continuous current response over the applied potential window without distinct redox peak features (Figure 5), which is characteristic of the predominantly capacitive/pseudocapacitive behavior of PEDOT:PSS-based electrodes (Kumar et al., 2015). The hysteresis between forward and reverse scans suggests the lack of electroactive biofilm and can further be attributed to limited (4 x 0.5 cm) surface area of anode. Nevertheless, the silver paint/surface-mount epoxy/titanium wire interface enabled charge transfer between the inkjet-printed PEDOT:PSS film and the external circuit after integration into BPV system. Electrochemical impedance spectroscopy provided additional supportive evidence of contact-associated resistance. Although the Nyquist response was noisy and yielded only an approximate equivalent circuit fit (Nyquist plot not shown here), the extracted Rs and Rct values of 95.28 Ω and 2.96 kΩ indicate that the interface remained electrically continuous but was still influenced by substantial interfacial resistive losses. Altogether, these analyses confirm that the selected interface was mechanically retained, electrically connected, and functionally operable under BPV conditions, while also highlighting scope for further optimisation of contact impedance.

Electrical interface stability is a key factor for stable and reliable operation of electrochemical systems and electronic devices (Huang et al., 2023). The practical application of a conductive component depends not only on its intrinsic electrical conductivity but also on the mechanical and electrical reliability of the interconnect joining it to the external circuit. Similarly, previous studies in polymer-based electrode systems have shown that conventional rigid metallic connectors or unsupported conductive adhesives often fail to provide durable contact when exposed to environmental and mechanical stress during prolonged operation (Li et al., 2019). This engineering principle is directly relevant to submerged BPV architectures, where the internal electrical interface is continuously challenged by moisture, ion penetration, adhesive weakening, and wire-induced stress concentration during operation. However, the interface stability and reliability of connection between anode and external circuit have largely been ignored. Therefore, the design of a BPV electrical interface need critical consideration and must be evaluated as a coupled mechanical-electrical junction rather than as a simple interconnection.

**Figure 6:**
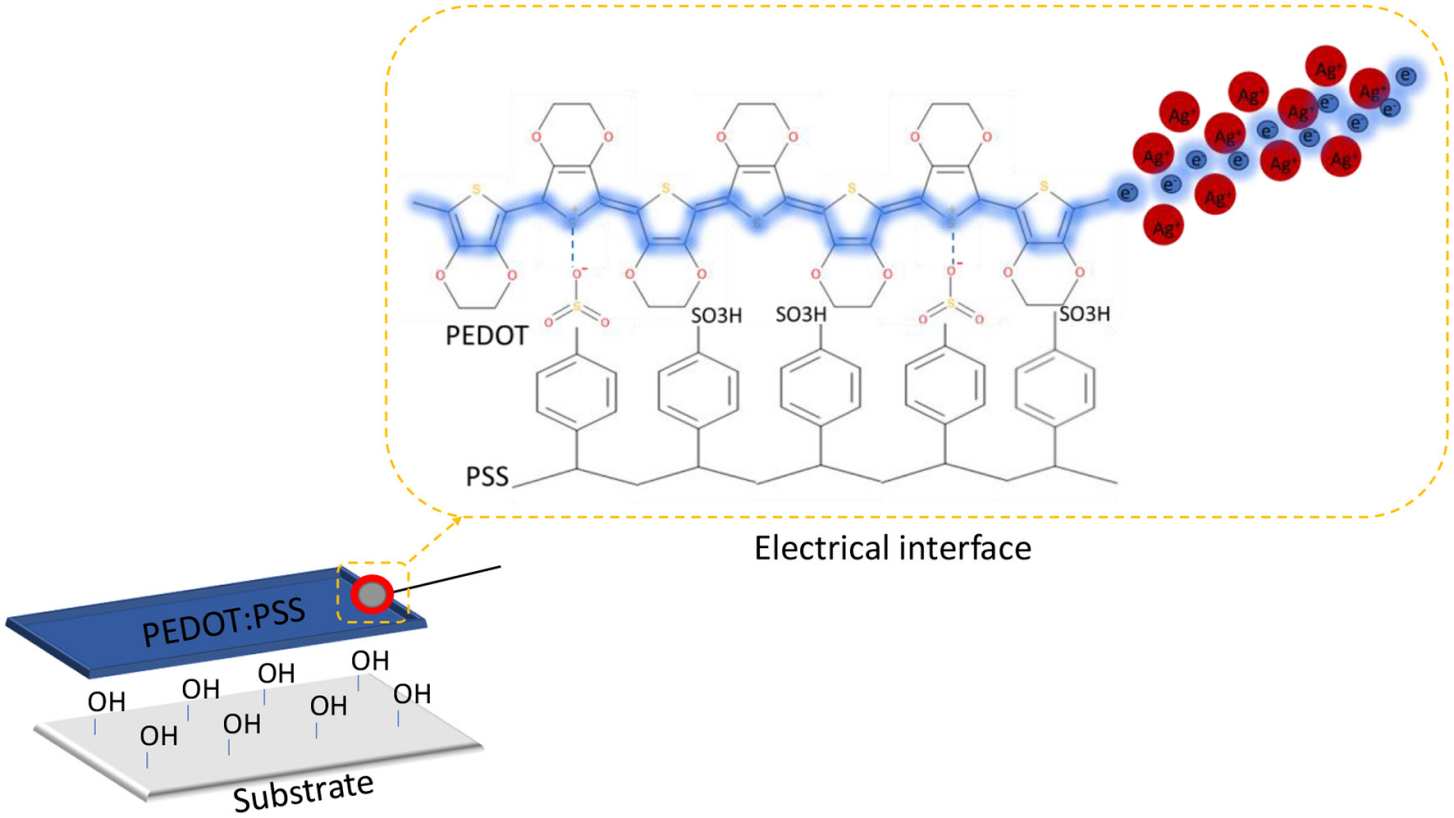
Schematic representation of inkjet-printed PEDOT:PSS anode and silver paint/surface-mount epoxy interface (Blue shading indicate path of electron transfer).

In this study, the long-term stability of the electrical interface was found to depend not only on the conductive adhesive itself but also on the adhesion behavior of the underlying substrate of inkjet-printed PEDOT: PSS film. A schematic representation of the final electrical interface configuration has therefore been included to clarify the spatial arrangement of the PEDOT: PSS layer, silver conductive paint, titanium wire, and epoxy overcoat (Figure 6). Although previous PEDOT:PSS film-only immersion studies showed comparatively superior film retention on plastic substrates (Anam et al., 2026), the complete silver paint/epoxy/titanium junction performed more consistently on UV-ozone treated glass, suggesting that overall interface survivability was governed not solely by PEDOT: PSS film adhesion but also by the rigidity of the supporting substrate and the anchoring behavior of the encapsulated wire contact.

PEDOT: PSS is known to undergo hydration and swelling in wet environments (Bießmann et al., 2018), which can progressively weaken weakly bonded interfaces and promote delayed delamination if stress is concentrated at the PEDOT:PSS/substrate boundary. In this study, the application of a rigid surface-mount epoxy overcoat improved junction durability by mechanically anchoring the wire beyond the conductive point contact, redistributing local stress, and reducing direct aqueous ingress into the silver/PEDOT:PSS region. These observations establish a clear structure–property relationship in which both substrate adhesion and epoxy encapsulation critically determine the mechanical retention and electrical functionality of submerged PEDOT:PSS-based BPV interfaces.

Few other limitations of the present study should be acknowledged here. The biological compatibility assessment was restricted to OD_750_-based cell growth monitoring and did not include detailed physiological or photosynthetic measurements. The interfacial electrical behavior needs to be further evaluated using more rigorous impedance analysis with higher fidelity fitting. Moreover, the sample size used in the screening tests was limited (*n* = 3), reflecting the preliminary comparative nature of this work. Although 7-day aqueous aging provided a useful short-term assessment of interface survivability, longer-term durability under continuous BPV operation remains to be investigated. These aspects warrant further study as the interface design progresses toward full device optimisation.

## 4 Conclusion

This study identifies the practical considerations for establishing electrical interface for inkjet-printed PEDOT:PSS anodes in biophotovoltaic (BPV) systems. Conventional mechanical interface connectors were found to be unsuitable due to the physical abrasion of the printed conductive film and corrosion under aqueous conditions, whereas the conductive adhesive interfaces required additional encapsulation to withstand prolonged immersion. Among the tested configurations, silver paint topped with a surface-mount epoxy overcoat provided the most robust combination of mechanical stability, electrical conductivity, preliminary biological compatibility, and BPV performance. The results further demonstrate that successful interface design is governed by coupled interactions between substrate surface properties, conductive contact, and protective encapsulation. Although further characterization of mechanical robustness and electrochemical investigations are required, the present work addresses an important and previously underexplored practical bottleneck in BPV electrode integration.

## Data Availability

The raw data supporting the conclusions of this article will be made available by the authors, without undue reservation.
