# Supplementary material for: Electrical interface design for additively manufactured polymer anodes for biophotovoltaic systems
Source: Front Microbiol. 2026 May 21;17:1858659. doi: 10.3389/fmicb.2026.1858659 (PMC13233488; doi:10.3389/fmicb.2026.1858659)
Supplement: Supplementary file 1 [file Table_1.docx]

**Supplementary material**

**Table S1:** Composition of BG11 growth media stock solution

| **Chemicals** | **Concentration (g/L)** | **Dilution factor** |
| --- | --- | --- |
| NaNO_3_ | 150 | 100 |
| K_2_HPO_4_ | 8 | 200 |
| MgSO_4_.7H_2_O | 15 | 200 |
| CaCl_2_.2H_2_O | 3.6 | 1000 |
| Trace elements | | 1000 |

**Table S2:** Composition of trace elements solution used in BG11 media

| **Chemicals** | **Concentration (g/L)** |
| --- | --- |
| H_3_BO_3_ | 2.86 |
| MnCl_2_.4H_2_O | 1.81 |
| ZnSO_4_.7H_2_O | 0.222 |
| NaMoO_4_.2H_2_O | 0.391 |
| CuSO_4_.5H_2_O | 0.079 |
| Co (NO_3_)_2_.6H_2_O | 0.049 |
| Citric acid | 6 |
| Ferric ammonium citrate | 6 |
| EDTA (disodium salt) | 1 |

**Table S3:** Composition of Bare conductive® electric paint (Bare Conductive Limited)

| **Component** | **Concentration (%)** |
| --- | --- |
| Water | 30-50 |
| Natural resin | 20-40 |
| Conductive carbon | 5-20 |
| Humectant | 5-10 |
| Processing aids and preservatives | 0-1 |

**Table S4:** Composition of Thermo Scientific^TM^ Carbon conductive cement adhesive

| **Component** | **Concentration (%)** | |
| --- | --- | --- |
| Proprietary non-hazardous ingredients | 25-50 | |
| Xylenes (o-, m-, p-isomers) | 10-25 |  |
| Methyl ethyl ketone | 10-25 | |
| Acetone | 10-25 | |
| Propylene glycol monomethyl ether acetate | 5-10 | |

**Table S5:** Composition of Silver conductive paint (RS PRO)

| **Composition** | **Concentration (%)** |
| --- | --- |
| Silver | ≥50 - ≤70 |
| 2-methylethyl acetate | ≥25 - ≤50 |
| n-butyl acetate | <5 |
| 2-butyoxyethyl acetate | <5 |

**Table S6:** Composition of RS PRO red syringe surface mount adhesive epoxy resin

| **Type** | **Component** | **Concentration (%)** |
| --- | --- | --- |
| **RS PRO red syringe surface mount adhesive** **epoxy resin** | Bisphenol-A-(epichlorhydrin) epoxy resin | 30-60 |
|  | 1,3-bis(2,3-epoxypropoxy)-2,2-dimethylpropane | 10-30 |
|  | Formaldehyde, oligomeric reaction products with phenol | 5-10 |
|  | Oxirane, mono[(C12-14-alkyloxy)methyl] derivs | <1 |
| **Flexible silicone coating** | Xylene | 30-60 |
|  | n-Butyl acetate | 5-10 |
|  | 2-octyl-2H-isothiazol-3-one | <1 |
| **Araldite® 2-part rapid epoxy** | 2,2'-[(1-methylethylidene)bis(4,1-phenyleneoxymethylene)]bisoxir ane | ≥70-<90 |
|  | 1,4-Bis(2,3- epoxypropoxy)butane | ≥3-<10 |
